# Quantitative analysis of anterior and posterior chamber parameters of primary angle-closure glaucoma based on level set method

**DOI:** 10.1186/s12886-026-04933-3

**Published:** 2026-05-22

**Authors:** Xin Wang, Yaru Yao, Xueyan Liu, Xinying You

**Affiliations:** 1 Department of Ophthalmology, Liaocheng People’s Hospital, Liaocheng, Shandong P.R. China; 2 School of Mathematical and Systems Science, Liaocheng University, Liaocheng, Shandong P.R. China; 3 Liaocheng Key Laboratory of Ophthalmology, Liaocheng People’s Hospital, Liaocheng, Shandong P.R. China

**Keywords:** Ultrasound biomicroscopy, Level set method, Intraocular pressure, Ocular parameters

## Abstract

**Purpose:**

The study aimed to accurately measure anterior and posterior chamber parameters in ultrasound biomicroscopy (UBM) images and to quantify their relationship with intraocular pressure (IOP), axial length (AL) and lens thickness (LT), enhancing the understanding of primary angle-closure disease.

**Methods:**

UBM images of 135 patients with primary angle-closure glaucoma(PACG) and 30 healthy individuals were analyzed. The Level set method was used to measure anterior chamber depth (ACD) and posterior chamber area (PCA). Relative error and intraclass correlation coefficient (ICC) were used to assess the accuracy and consistency of these measurements compared to the ground truth. IOP, AL and LT were also measured, and the relationships between ACD, PCA, IOP, AL and LT were explored using Spearman correlation analysis.

**Results:**

The Level set method yielded highly accurate measurements of ACD and PCA with low Relative errors and high ICC values. Spearman correlation analysis revealed that PCA was significantly positively correlated with both AL (*R* = 0.398, *p* = 0.002) and LT (*R* = 0.255, *p* = 0.006). In contrast, no statistically or clinically significant associations were found for the remaining parameter pairs (e.g., ACD–IOP, PCA–IOP), all of which showed |R|<0.2 and *P* > 0.05.

**Conclusion:**

The Level set method proves to be a highly accurate and consistent technique for UBM image analysis, boosting measurement efficiency and reproducibility. The significant correlations of PCA with AL and LT offer new insights into the anatomical complexity of angle-closure disease, emphasizing the posterior chamber as a critical dimension for further exploration.

## Introduction

Glaucoma is the leading cause of irreversible blindness globally, with an estimated 111.8 million patients by 2040 [1]. Studies have shown that primary angle-closure glaucoma (PACG) has a higher prevalence in Asian populations, particularly among East Asians [2]. In China, glaucoma patients account for a quarter of the global total, with 9.4 million patients over the age of 40 [3]. As China enters an aging society rapidly, glaucoma has become a significant public health issue.

Therefore, further research into the pathogenesis of PACG is of utmost importance in China [4]. PACG is closely correlated with the anatomical structure of the eye, particularly the following biometric characteristics: shallow anterior chamber depth (ACD), increased lens thickness(LT), and short axial length (AL) [5]. Currently, research on anterior segment parameters in PACG has been relatively well-established, with significant progress achieved particularly in the assessment of indicators such as anterior chamber depth and angle width, gradually advancing toward personalized diagnosis and treatment [6]. However, as an important component of the anterior segment, the specific role of morphological changes in the posterior chamber in the pathogenesis of PACG remains to be further elucidated.

Existing studies have demonstrated that the posterior chamber plays an indispensable role in the onset and progression of PACG. Quigley et al. pointed out that pressure imbalance between the anterior and posterior chambers serves as the core kinetic mechanism underlying relative pupillary block, suggesting a close association between posterior chamber status and PACG pathogenesis [7]. Zheng et al. further revealed that abnormalities in the lens-zonule posterior space play a significant role in the development of both PACG and malignant glaucoma [8]. In their proposed personalized diagnostic and therapeutic system for PACG, Kurysheva et al. emphasized that integrating visualization techniques with data-driven machine learning analysis of anterior and posterior segment structures facilitates precise phenotyping based on different pathogenic mechanisms and enables personalized interventions, aligning with the core principles of predictive, preventive, and personalized medicine (“3P Medicine”) [6]. Therefore, this study focuses on the precise quantitative analysis of posterior chamber parameters, aiming to explore an effective and reliable method for posterior chamber assessment, thereby providing a basis for elucidating the role of posterior chamber structures in the pathogenesis of PACG.

Although ophthalmic imaging technology has advanced rapidly, techniques for imaging and analyzing the posterior chamber remain relatively underdeveloped. Although Anterior Segment Optical Coherence Tomography(AS-OCT) offers high-resolution images of the angle, iris, and lens, it is relatively limited in studying the posterior chamber structure, especially when evaluating structures other than scleral spur parameters [9]. Ultrasound Biomicroscopy (UBM) provides clear images of both the anterior and posterior chambers, which is of great value in the study of PACG [10]. However, the irregular and complex boundaries of the posterior chamber in UBM images pose a significant challenge to traditional manual analysis methods [11].

Recent advancements in computer technology have enabled automated analysis of medical images using computer-aided image processing techniques [12]. This technology has the advantages of time-saving, high efficiency, ease of use, and high consistency. In particular, the Level set method, as a powerful image segmentation technique, has demonstrated significant advantages in addressing image intensity inhomogeneity and improving segmentation accuracy [13]. When combined with local intensity clustering and edge-based segmentation techniques, this method has achieved outstanding results in various medical imaging fields [14–17], such as breast MRI and prostate ultrasound [18–20]. In ophthalmology, the Level set method has also shown excellent performance in analyzing complex ocular structures, such as Schlemm’s canal and the trabecular meshwork, laying a solid foundation for its application in UBM image processing [21,22]. These successes highlight its potential for enhancing UBM image processing, promising better measurement accuracy for the anterior and posterior chambers.

This study innovatively applies the Level set method to UBM image analysis, enabling precise segmentation and measurement of ACD and posterior chamber area (PCA) in PACG patients. By analyzing the correlations between anterior chamber and posterior chamber parameters with intraocular pressure(IOP), AL and LT, it provides novel insights into the pathogenesis of angle-closure glaucoma.

## Methods

### Image acquisition

According to the inclusion and exclusion criteria, patients with Acute Primary Angle Closure Attack (APACA) were recruited from the glaucoma department of Liaocheng People’s Hospital between January 2021 and December 2023 [23,24], with the acutely affected eye selected as the study subject. Healthy control data were obtained from age-matched uninjured eyes of trauma patients undergoing UBM examination, with ocular normality confirmed per our established criteria [21]. The main inclusion criteria for the patient group were: age > 40 years, acute angle closure, characteristic symptoms and clinical signs of APACA, reduction in IOP and alleviation of symptoms following medication. The main inclusion criteria for the control group were: age > 40 years, IOP 10–21 mmHg; normal anterior chamber depth, and normal angle structure confirmed by gonioscopy. The main exclusion criteria were: history of ocular trauma; ocular laser treatment or surgery in the study eye; presence of other ocular diseases; high myopia or hyperopia with a spherical equivalent refrative error (greater than + 3 or − 3 diopters); poor image quality preventing further analysis; inability to cooperate with the examination; inability to understand and sign the informed consent form. All subjects underwent detailed eye examinations, including visual acuity, optometry (Topcon auto refractometer RM-1, Tokyo, Japan), slit-lamp microscopy, fundus examination, IOP measurement (iCare TA01i Rebound Tonometer, iCare Finland Oy), and gonioscopy. The patient group additionally underwent visual field examination (Humphrey Visual Field AnalyzerII, Carl Zeiss Meditec, Dublin, USA) and axial length and lens thickness measurements by A-scan (Quantel Medical AVISO Ophthalmic A/B-Scan Ultrasound System, Clermont-Ferrand, France). A 50 MHz UBM (Suoer SW-3200 L, Suowei Co., Tianjin, China) was used for image acquisition of the anterior and posterior chamber in all subjects. All examinations were conducted by the same ophthalmologists under the same illumination conditions using the same equipment.

### Level set method

The level set method, widely used in medical image segmentation [25], was previously applied to segment the trabecular meshwork and Schlemm’s canal in healthy UBM images [21]. Our study extends this approach to glaucoma patients’ anterior and posterior chamber segmentation, addressing greater challenges from structural deformations. We optimized parameters for robust performance: a centered 30 × 40-pixel initial contour, distance regularization (µ = 1.0), neighborhood scale (σ = 4), smoothness coefficient (λ = 1.0), iteration limits (outer = 500, inner = 10), and convergence threshold (1 × 10^− 3^). These empirically validated settings ensured precise boundary delineation while efficiently handling glaucoma’s higher anatomical variability.

Using ImageJ software, each image was segmented three times by the same ophthalmologist, with the average of these measurements considered as the ground truth. We conducted a comprehensive evaluation of the Level set method’s accuracy and repeatability in measuring ACD and PCA. First, we quantified geometric discrepancies through relative error analysis, followed by validation against the ground truth using paired t-tests [26]. To further assess measurement consistency, we performed intraclass correlation coefficient (ICC) analysis on repeated segmentations [27]. Building upon these methodological validations, we then employed Spearman correlation analysis to investigate potential relationships between four clinically relevant ocular parameters - ACD, PCA, IOP, and AL - all derived from our Level set segmentation results [28].

## Results

### Image segmentation and parameter measurement

**Fig. 1:**
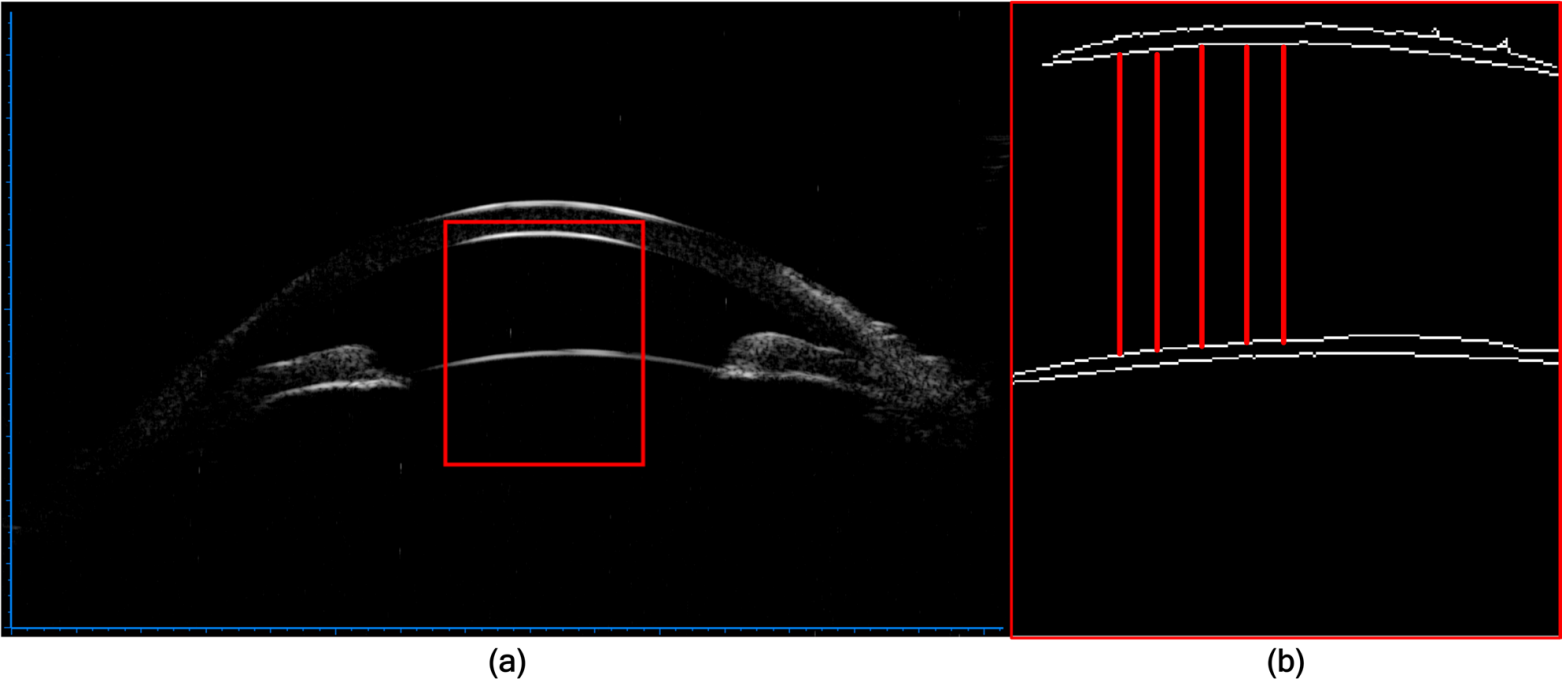
(**a**) Region of interest in the anterior chamber, (**b**) Method for calculating its depth

As shown in Fig. 1, the ACD in UBM images can be quantified through the following steps:

Region of interest (ROI) selection: Define the ROI, encompassing the corneal endothelium and the anterior lens capsule.

Boundary Detection: Apply the Level set method to identify the boundaries of the corneal endothelium and lens capsule within the ROI.

Distance Calculation: Measure the vertical distance (in pixels) between the two boundaries column by column from left to right.

Averaging: Select the five largest measured distances and compute their mean as the final ACD value.

This automated method ensures high precision in ACD measurement, eliminating manual measurement errors.

**Fig. 2:**
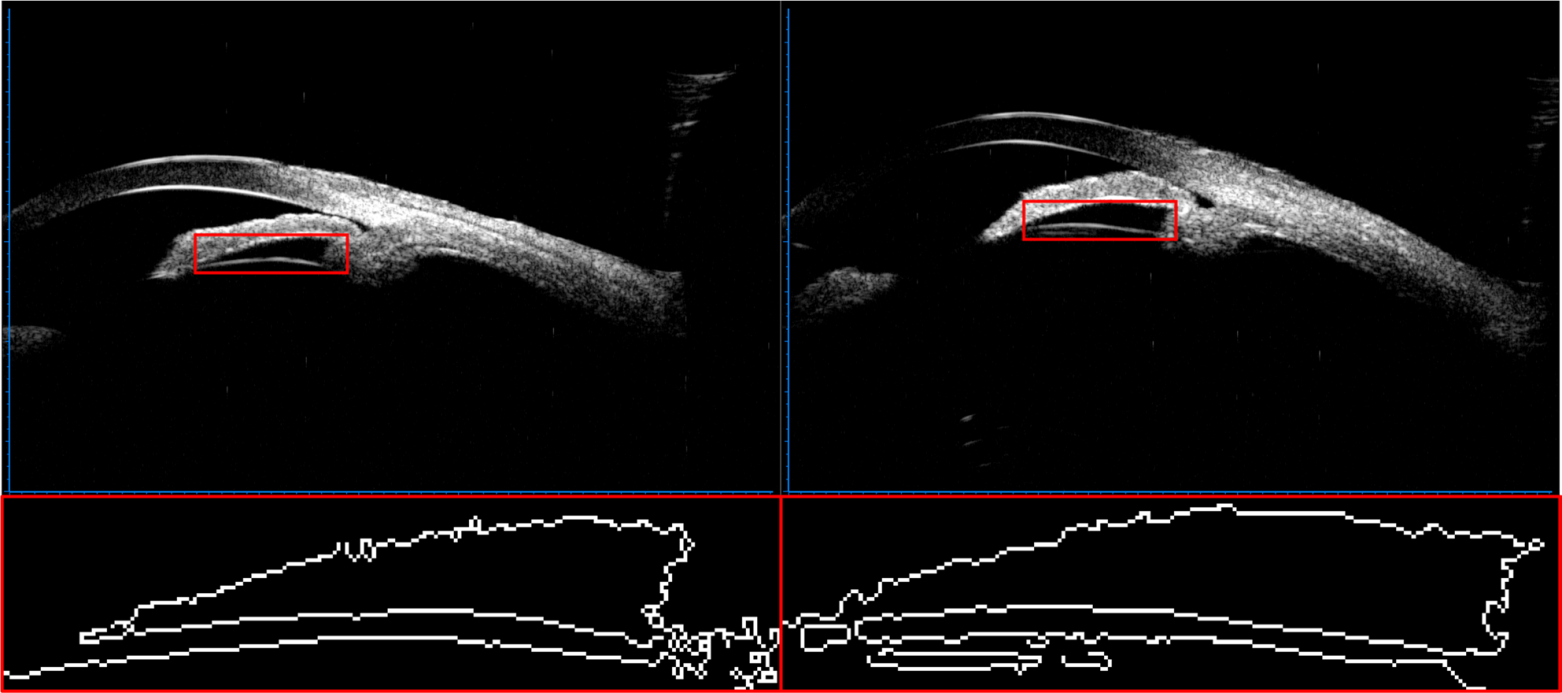
The first row displays two UBM images depicting complete posterior chamber boundaries and their respective ROI. The second row illustrates the segmented boundary of the ROI within the posterior chamber

As indicated by the rectangular box in Fig. 2, the posterior chamber is defined as the annular space bounded by the posterior iris surface, inner ciliary body, anterior lens capsule, and zonular fibers. The area measurement procedure consists of the following steps:

ROI Selection: The ROI is identified in the UBM image, encompassing the posterior iris surface, inner ciliary body, anterior lens capsule, and zonular fibers.

Boundary Detection: The Level set method is employed to segment the UBM image and delineate the boundaries of the posterior chamber.

Distance Calculation: For each image column of the boundary contour, the number of pixels between the two boundary curves is computed.

Area Summation: The total PCA is obtained by summing the pixel counts across all columns.

The level set method enables objective quantification of PCA through precise boundary extraction, effectively eliminating manual measurement bias and demonstrating particular efficacy in analyzing complex, irregular ocular structures.

**Fig. 3:**
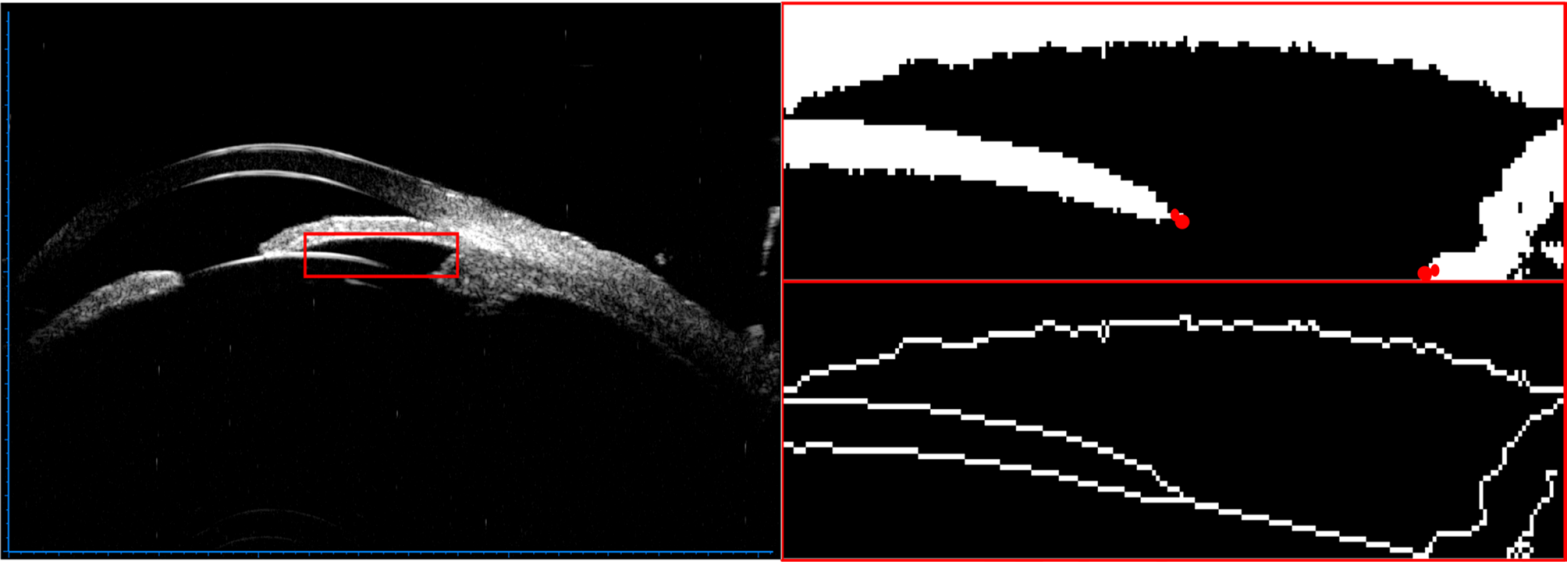
Binary images of the posterior chamber and its boundaries in UBM images. When the posterior chamber boundary is incomplete, it is defined by extending the suspensory ligament to its intersection with the anterior lens surface

However, incomplete boundaries are observed in the posterior chamber of UBM images. As shown in the left image of Fig. 3, the absence of zonular fibers results in discontinuous posterior chamber boundaries. To address this issue, we propose a boundary completion strategy: first, the UBM image is segmented using the Level set method, followed by connecting the breakpoints of the lens with the ciliary processes to generate a smooth curve. This curve serves as the boundary of the ROI for the posterior chamber, effectively compensating for the missing boundary segments. The proposed method successfully resolves the boundary discontinuity issue, providing a reliable data foundation for subsequent quantitative analysis.

### Quantitative validation of results based on Level set method

**Fig. 4:**
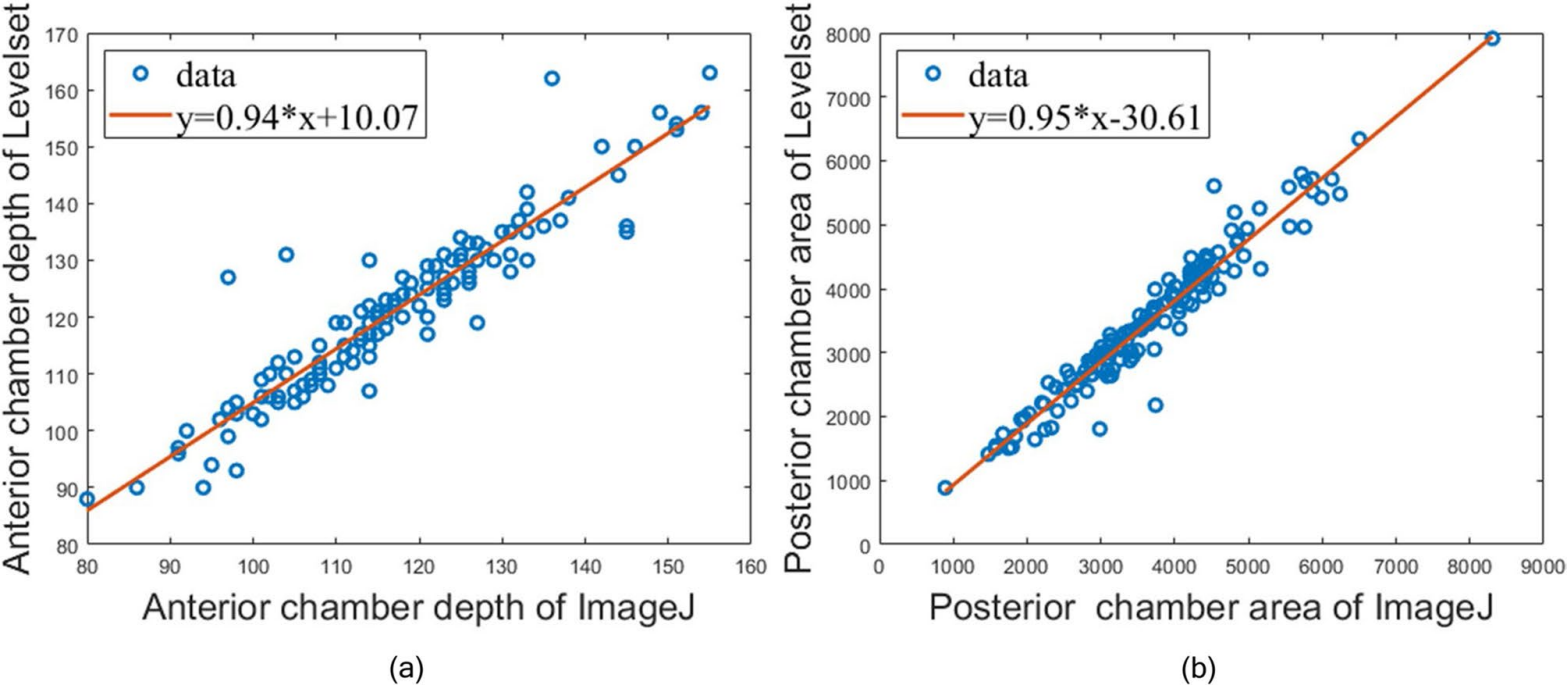
The comparative and correlation analysis chart of two methods for measuring anterior chamber depth and posterior chamber area

Accurate measurement of the ACD and the PCA plays a crucial role in the diagnosis of ophthalmic diseases. In this subsection, we evaluate the performance of the Level set method in measuring the ACD and PCA. Compared with the ImageJ ground truth, this method demonstrated excellent measurement consistency. Scatter plot analysis showed that the fitted slopes of ACD and PCA were 0.94 and 0.95 respectively (Fig. 4). The data points were closely distributed around the fitted lines, indicating a high degree of agreement in the measurement results.

Quantitative analysis confirmed the reliability of this method. The relative errors of ACD and PCA were both lower than 0.017 (0.0165 and 0.0085). The paired t - test showed no significant differences (*p* = 0.475 and 0.839), and the ICC were both greater than 0.99 (0.990 and 0.995). These results indicate that the level set method has clinical - grade measurement accuracy and repeatability, providing a reliable automated measurement tool for the diagnosis of ophthalmic diseases.

### Quantitative comparative evaluation of ocular physiological parameters between patients and healthy control group

**Table 1:** Mean ± standard deviation (pixels) of UBM biometric values obtained from PACG patients and healthy control group

	Method	ACD	PCA
PACG patients	ImageJ	116.88 ± 14.61	3488.94 ± 1173.9
	Level set	120.94 ± 14.79	3665.45 ± 1184.57
healthy control	ImageJ	197.47 ± 17.27	4142.97 ± 981.12
	Level set	199.30 ± 16.97	4192.87 ± 958.39

This study measured the ACD and PCA in 135 PACG patients and 30 healthy controls. The measurement data were presented in pixels, and the specific values are shown in Table 1. The results showed that the Level set method consistently yielded slightly higher measurements for both ACD and PCA compared to the ImageJ method. Although this trend was observed in both groups, the differences were smaller in the control group, indicating that the Level set method maintains good measurement stability in normal ocular structures while still exhibiting a slight tendency for overestimation. The slight overestimation observed in the Level Set method relative to manual tracing, especially in normal eyes, likely stems from the conservative bias of human operators when delineating low-contrast boundaries (e.g., the iris-lens interface). While humans tend to under-segment to avoid inclusion errors, the algorithm’s gradient-driven evolution captures sub-pixel anatomical details more comprehensively, suggesting that the automated measurements may actually approximate the true anatomical extent more accurately than manual tracing in these ambiguous regions.

**Fig. 5:**
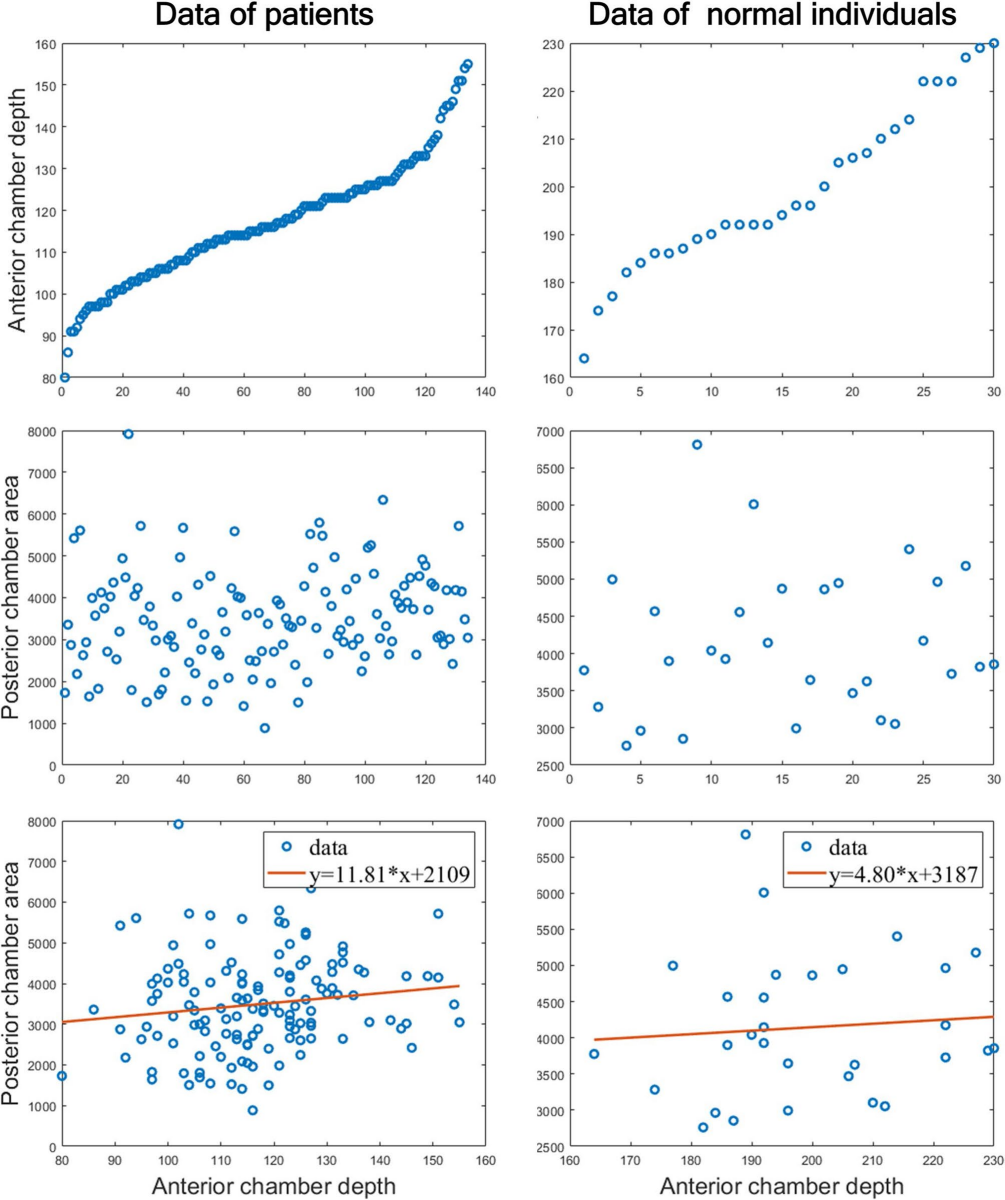
Comparison of the relationship between ACD and PCA between PACG patients and healthy control group

Figure 5 clearly demonstrates significant differences in ACD and PCA measurements between PACG patients and healthy controls. PACG patients showed markedly shallower ACD (80–160 pixels) compared to controls (160–230 pixels). PCA measurements were more variable in patients (1000–8000 pixels) than in controls (2500–7000 pixels).Regression analysis revealed distinct patterns: PACG patients exhibited a steeper PCA growth rate (slope = 11.81) with increasing ACD, while controls showed a gentler trend (slope = 4.80). This contrast highlights anatomical differences between the groups.

### Correlation analysis between parameters

We used Spearman correlation analysis to evaluate the correlations among five ophthalmic parameters in patients with PACG. The results are shown in Table 2.

**Table 2:** Spearman correlation coefficient matrix of ophthalmic biometric indicators (*N* = 135)

	PCA	ACD	IOP	AL
ACD	0.165	1		
IOP	-0.09	-0.124	1	
AL	0.398**	0.183	0.029	1
LT	0.255**	-0.106	-0.054	0.038

** *p* < 0.01

According to the Spearman correlation analysis, PCA was significantly positively correlated with AL (*R* = 0.398, *p* = 0.002) and LT (*R* = 0.255, *p* = 0.006). This finding suggests that in ophthalmic diagnosis, PCA may increase with axial elongation and lens thickening. In contrast, the correlations between IOP and PCA, ACD, AL, and LT did not reach statistical significance (*p* = 0.337, 0.187, 0.761, and 0.564, respectively, with absolute correlation coefficients all below 0.13).

## Discussion

This study applied the level set algorithm for automated analysis of UBM images from patients with PACG to precisely quantify ACD and PCA, and explored their correlations with IOP, AL and LT. The level set method demonstrated accurate automated segmentation and measurement of anterior and posterior chamber structures in UBM images, exhibiting excellent accuracy and reproducibility in measuring ACD and PCA. Compared with the manual method, the ICC for this method was 0.99, with relative errors maintained at a low level, confirming its reliability for clinical-grade applications. This provides robust support for the consistency and efficiency of data acquisition in future large-scale clinical studies.

The most insightful finding of this study is the significant positive correlation of PCA with both AL and LT, which expands the research perspective on the posterior chamber. Classical theories suggest that PACG is primarily associated with shallow anterior chamber, thick lens, and short axial length. This study partially supports this view, as ACD was significantly shallower in PACG patients compared to the healthy control group. However, the positive correlation between PCA and AL suggests that in the eyes with relatively longer axial lengths, the posterior chamber space may be larger. The potential mechanism might involve axial elongation accompanied by changes in ciliary body position and zonular tension, thereby influencing the spatial configuration of the posterior iris surface. The relationship between PCA and LT appears more complex. Theoretically, lens thickening should push the iris forward and compress the posterior chamber. Yet, this study found a positive correlation between them. We hypothesize that while lens volume increases, the contact area or relative position between the lens and the posterior iris surface may undergo compensatory adjustments, ultimately leading to a net increase in posterior chamber space. This suggests that the mechanism of angle closure is not merely anterior chamber “crowding” but rather the result of an imbalance in the spatial relationships among the anterior chamber, posterior chamber, lens, and axial length.

This study did not find significant correlations between IOP and ACD, PCA, AL or LT. This negative result requires cautious interpretation, primarily because all enrolled PACG patients were receiving IOP-lowering medications. Pharmacological treatment reduced IOP, potentially decoupling it from the natural correspondence with original anatomical structures, thereby diluting potential associations. Due to ethical considerations and patient safety concerns, obtaining UBM images in an unmedicated state during acute angle-closure attacks—the critical window for observing direct associations between anatomical structures and pathological high IOP—was not feasible. Therefore, this finding does not negate the role of anatomical factors in IOP elevation but rather emphasizes that in treated populations, IOP can no longer serve as a reliable indicator of baseline anatomical risk.

In addition to these findings, several limitations of this study should be acknowledged. First, as a cross-sectional study, we can only reveal parameter associations and cannot infer causality. Second, the sample size was relatively small, particularly for the control group. Third, the zonules are three-dimensional structures, whereas UBM captures two-dimensional images; thus, quantitative analysis of posterior chamber area may have some inherent bias. Future research could advance in the following directions: (1) Conduct prospective studies to establish baseline anatomical parameters and their long-term associations with IOP in high-risk populations or newly diagnosed, untreated patients; (2) Increase sample diversity, including different subtypes of primary angle closure disease to validate the algorithm’s generalizability; (3) Utilize the level set method to longitudinally observe dynamic changes in anterior and posterior chamber parameters before and after surgical or laser treatments.

## Conclusion

This study validated the clinical utility of the level set method as a precise and efficient tool for analyzing anterior and posterior chambers in UBM images. Quantitative analysis revealed significant positive correlations between PCA and both AL and LT. This finding enriches our understanding of the complex anatomical basis of angle-closure disease and suggests that posterior chamber morphology is a dimension worthy of further exploration.

## Data Availability

The data that support the findings of this study are available from the corresponding author upon reasonable request.

## Abbreviations

UBM: Ultrasound Biomicroscopy
IOP: Intraocular Pressure
AL: Axial Length
LT: Lens Thickness
PACG: Primary Angle-Closure Glaucoma
ACD: Anterior Chamber Depth
PCA: Posterior Chamber Area
ICC: Relative error and intraclass Correlation Coefficient
AS-OCT: Anterior Segment Optical Coherence Tomography
APACA: Acute Primary Angle Closure Attack
ROI: Region Of Interest
